# Comprehensive Analysis Identifies the PPAR-Targeted Genes Associated with Ovarian Cancer Prognosis and Tumor Microenvironment

**DOI:** 10.1155/2023/6637414

**Published:** 2023-05-11

**Authors:** Xiao-Fei Leng, Gao-Fa Wang, Hao Yin, Feng Wei, Kang-Kang Zeng, Yi-Qun Zhang

**Affiliations:** ^1^Department of Obstetrics and Gynecology, Taihe Hospital, Hubei University of Medicine, Shiyan, China; ^2^State Key Laboratory of Ultrasound in Medicine and Engineering, Chongqing Medical University, Chongqing 400016, China; ^3^Department of Gynecologic Oncology, Beijing Obstetrics and Gynecology Hospital, Capital Medical University, No. 251, Yaojiayuan Road, Chaoyang District, Beijing, China

## Abstract

**Background:**

There is a significant role for peroxisome proliferator-activated receptors (PPARs) in the development of cancer. Nevertheless, the role of PPARs-related genes in ovarian cancer (OC) remains unclear.

**Methods:**

The open-accessed data used for analysis were downloaded from The Cancer Genome Atlas database, which was analyzed using the R software.

**Results:**

In our study, we comprehensively investigated the PPAR target genes in OC, including their biological role. Meanwhile, a prognosis signature consisting of eight PPAR target genes was established, including apolipoprotein A-V, UDP glucuronosyltransferase 2 family, polypeptide B4, TSC22 domain family, member 1, growth hormone inducible transmembrane protein, renin, dedicator of cytokinesis 4, enoyl CoA hydratase 1, peroxisomal (ECH1), and angiopoietin-like 4, which showed a good prediction efficiency. A nomogram was constructed by combining the clinical feature and risk score. Immune infiltration and biological enrichment analysis were applied to investigate the difference between high- and low-risk patients. Immunotherapy analysis indicated that low-risk patients might respond better to immunotherapy. Drug sensitivity analysis indicated that high-risk patients might respond better to bleomycin, nilotinib, pazopanib, pyrimethamine, and vinorelbine, yet worse to cisplatin and gefitinib. Furthermore, the gene ECH1 was selected for further analysis.

**Conclusions:**

Our study identified a prognosis signature that could effectively indicates patients survival. Meanwhile, our study can provide the direction for future studies focused on the PPARs in OC.

## 1. Introduction

Around the world, ovarian cancer (OC) remains one of the most lethal gynecological cancers [1]. With high mortality, the incidence rate of OC still shows an upward trend, making it a serious public health threat [2]. Nowadays, surgery and chemotherapy are the main treatments for OC. Meanwhile, as a result of hidden early symptoms, many patients have entered the progressive stage of the disease after their first diagnosis, missing the best time for treatment [3]. Consequently, exploring new targets with potential for clinical application is extremely important [4].

Peroxisome proliferator-activated receptors (PPARs) are a kind of nuclear receptors regulated by ligands and are involved in sensing nutrients, regulating metabolism, and regulating lipids [5]. Considering the wide regulatory effect of PPARs, researchers have begun paying attention to their role in a variety of diseases, especially in cancers [5]. Yang et al. found that the interaction between PPAR*γ* and Nur77 can contribute to fatty acid uptake, therefore, promoting breast cancer development [6]. Zou et al. noticed that the PPAR*γ* signaling could be activated by the polyunsaturated fatty acids from astrocytes, further facilitating the brain metastasis process of cancer [7]. Moreover, PPARs signaling is associated with the immune cells in cancer tissue. Liu et al. indicated that S100A4 could regulate the fatty acid oxidation dependent on PPAR*γ* and, therefore, induce M2 polarization in cancer [8]. Furthermore, various pieces of evidence indicate that cancer cells up-regulated PPAR*δ*, which can be used as a defense mechanism against nutritional deprivation and energy stress to improve its survival rate and promote cancer progression [9]. In OC, some studies have preliminarily explored the potential mechanism of PPARs [10]. However, there are still few studies focusing on PPAR in OC.

In recent years, the development of bioinformatics is accompanied by the arrival of the big data era, which provides convenience for researchers [11–13]. In our study, we comprehensively investigated the PPAR target genes in OC, including their biological role. Meanwhile, a prognosis signature consisting of eight PPAR target genes was established, including apolipoprotein A-V (APOA5), UDP glucuronosyltransferase 2 family, polypeptide B4 (UGT2B4), TSC22 domain family, member 1 (TSC22D1), growth hormone inducible transmembrane protein (GHITM), renin (REN), dedicator of cytokinesis 4 (DOCK4), enoyl CoA hydratase 1, peroxisomal (ECH1), and angiopoietin-like 4 (ANGPTL4). Immune infiltration and biological enrichment analysis were applied to investigate the difference between high- and low-risk patients. Immunotherapy and drug sensitivity analysis were then conducted. Furthermore, the gene ECH1 was selected for further analysis.

## 2. Methods

### 2.1. Acquisition of Open-Accessed Data

The expression profile and clinical characteristics of OC patients were downloaded from The Cancer Genome Atlas Program (TCGA) database (TCGA-OV project). The individual file was merged using the R code. Data pre-processing was conducted before the analysis. The list of 126 PPAR target genes was obtained from the PPARgene database (Supplementary Table S1) [14]. The baseline information of enrolled patients was shown in Table 1.

### 2.2. Biological Difference Investigation

Clusterprofiler was used in the R environment to perform Gene Ontology (GO) and Kyoto Encyclopedia of Genes and Genomes (KEGG) analysis [15]. Gene Set Enrichment Analysis (GSEA) was performed to identify the biological differences based on the specific gene set, including Hallmark and GO [16].

### 2.3. Prognosis Signature

First, patients were randomly divided into the training group and validation group according to the ratio of 1 : 1. Univariate Cox regression analysis was performed to identify the genes closely related with patients survival. The Least absolute shrinkage and selection operator (LASSO) regression algorithm was applied to screen the optimized variables through data dimension reduction. Ultimately, the multivariate Cox regression was utilized to identify a prognosis signature.

### 2.4. Model Evaluation and Nomogram

The performance of identified prognosis signature was completed using the Kaplan–Meier (KM) and receiver operating characteristic (ROC) curves. A nomogram combining our prognosis signature and clinical features was established using the rms package. The calibration curve was used to compare the fit between nomogram-predicted and actual survival.

### 2.5. Immune Infiltration and Function Analysis

The quantification of the OC tumor microenvironment was evaluated using multiple algorithms, including CIBERSORT, EPIC, MCP-counter, quanTIseq, TIMER, and xCell [17]. The expression profile of OC patients was set as the input file. Immune function analysis was performed based on the single sample GSEA (ssGSEA) algorithm [18].

### 2.6. Evaluation of Immunotherapy and Drug Sensitivity

The assessment of patients on immunotherapy response was performed using the Tumor Immune Dysfunction and Exclusion (TIDE) algorithm [19]. Drug sensitivity analysis was conducted based on the data from the Genomics of Drug Sensitivity in Cancer database [20].

### 2.7. Statistical Analysis

Analysis based on public data was all analyzed using the R software. The threshold of statistical significance was set as 0.05. Different statistical methods are selected according to different data distribution forms. The data with normal distribution were analyzed using the Students *T* test, and the non-normal distribution data was analyzed using the Mann–Whitney *U* test.

## 3. Results

**Figure 1:**
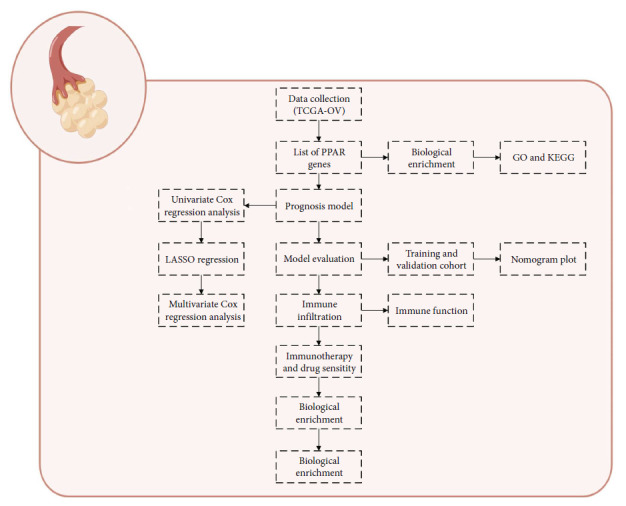
The flow chart of whole study.

The flow chart of the whole study was shown in Figure 1.

### 3.1. Collection of PPAR Target Genes in OC

**Figure 2:**
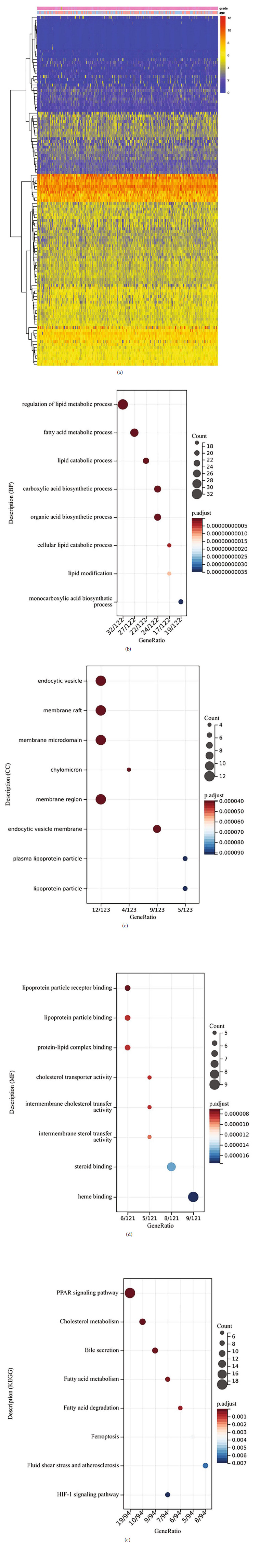
Role of PPAR target genes in OC. (a) The expression pattern of PPAR target genes in OC. (b) GO-BP analysis of these PPAR target genes. (c) GO-CC analysis of these PPAR target genes. (d) GO-MF analysis of these PPAR target genes. (e) KEGG analysis of these PPAR target genes.

First, the expression data of 126 PPAR targets were extracted, which was shown in Figure 2(a). Results of GO-Biological Process (BP) showed that the regulation of the lipid catabolic process, lipid metabolic process, and carboxylic acid biosynthetic process were top enriched terms of these genes (Figure 2(b)). For the GO-Cell Component (CC), these genes were primarily enriched in the endocytic vesicle, membrane raft, membrane microdomain, and chylomicron (Figure 2(c)). For the GO-Molecular Function (MF), these genes were mainly enriched in lipoprotein particle receptor binding, lipoprotein particle binding, protein–lipid complex binding, and cholesterol-transported activity (Figure 2(d)). For the KEGG analysis, these genes were mainly enriched in the PPAR signaling pathway, cholesterol metabolism, bile secretion, and fatty acid metabolism (Figure 2(e)).

### 3.2. Identification of a Prognosis Signature Robustly Indicating Patients Survival

**Figure 3:**
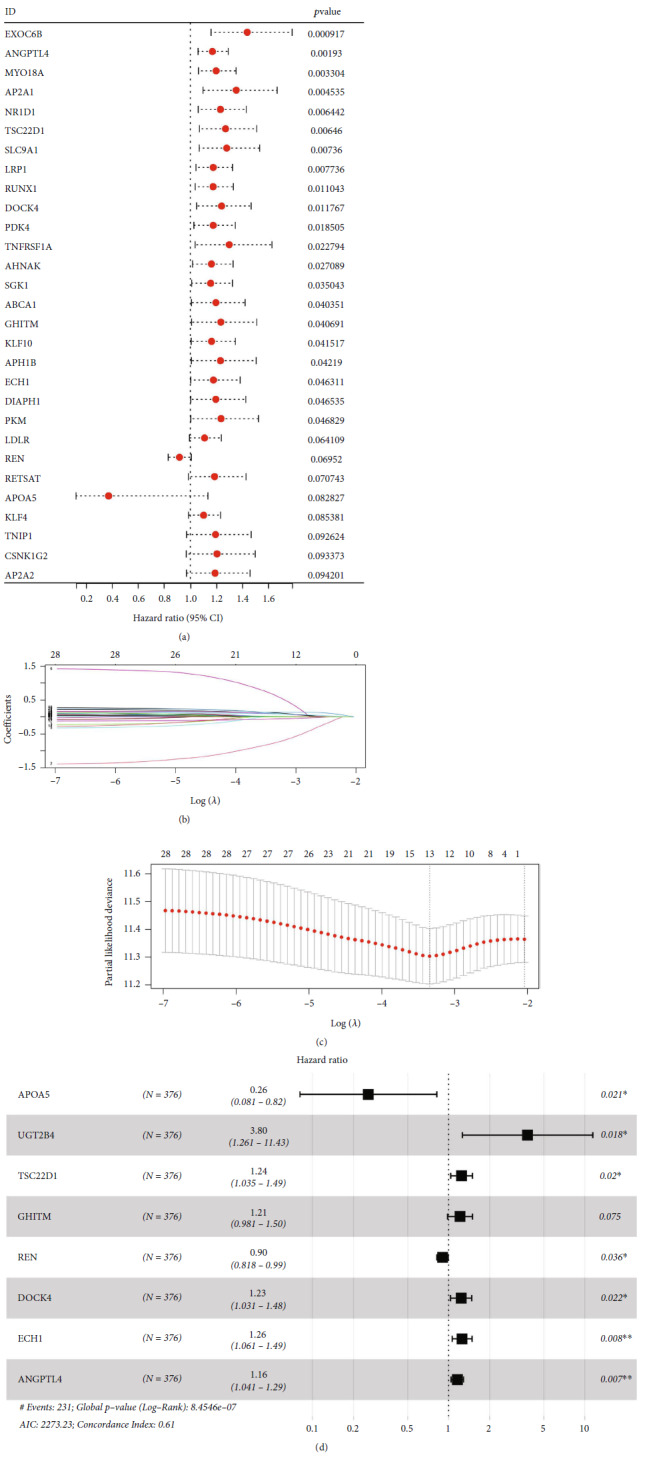
Identification a prognosis signature based on the PPAR target genes. (a) Univariate Cox regression analysis was performed to identify the prognosis-related genes. (b and c) LASSO regression analysis. (d) Multivariate Cox regression analysis was utilized to identify the prognosis signature.

Then, based on these PPAR target genes, the univariate Cox regression analysis was utilized to identify the genes close to patients survival with *P* < 0.1 (Figure 3(a)). Subsequently, the LASSO regression analysis was utilized to screen the optimized variables through data dimension reduction (Figures 3(b) and 3(c)). Finally, multivariate Cox regression analysis identified a prognosis signature consisting of eight PPAR target genes, including APOA5, UGT2B4, TSC22D1, GHITM, REN, DOCK4, ECH1, and ANGPTL4 (Figure 3(d)). The formula of “Risk score = APOA5 × −1.358 + UGT2B4 × 1.334 + TSC22D1 × 0.218 + GHITM × 0.192 + REN × −0.134 + DOCK4 × 0.211 + ECH1 × 0.228 + ANGPTL4 × 0.146” was utilized to calculate the risk score. The median value of risk score was used to divide high- and low-risk patients. The biological enrichment analysis of these model genes was shown in Figure S1. Results indicated that for the patients with high ANGPTL4 expression, the top 3 enriched pathways were angiogenesis, cholesterol hemoostasis, and interleukin 6/Janus kinase/signal transducer and activator of transcription 3 signaling (Figure S1(a)); for the patients with high APOA5 expression, the top 3 enriched pathways were V-Ki-ras2 Kirsten ratsarcoma viral oncogene homolog (KRAS) signaling, spermatogenesis, and pancreatic beta cells (Figure S1(b)); for the patients with high DOCK4 expression, the top 3 enriched pathways were angiogenesis, hedgehog signaling, and transforming growth factor-beta signaling (Figure S1(c)); for the patients with high ECH1 expression, the top 3 enriched pathways were KRAS signaling DN, E2F targets, and G2M checkpoint (Figure S1(d)); for the patients with high GHITM expression, the top 3 enriched pathways were reactive oxygen species pathway, MYC targets, and cholesterol homeostasis (Figure S1(e)); for the patients with high REN expression, the top 3 enriched pathways were estrogen response late, KRAS signaling, and G2M checkpoint (Figure S1(f)); for the patients with high TSC22D1 expression, the top 3 enriched pathways were angiogenesis, hedgehog signaling, and Wnt/*β*-catenin signaling (Figure S1(g)); and for the patients with high UGT2B4 expression, the top 3 enriched pathways were epithelial–mesenchymal transition (EMT), mitotic spindle, and ultraviolet (UV) response DN (Figure S1(h)).

### 3.3. Model Evaluation

**Figure 4:**
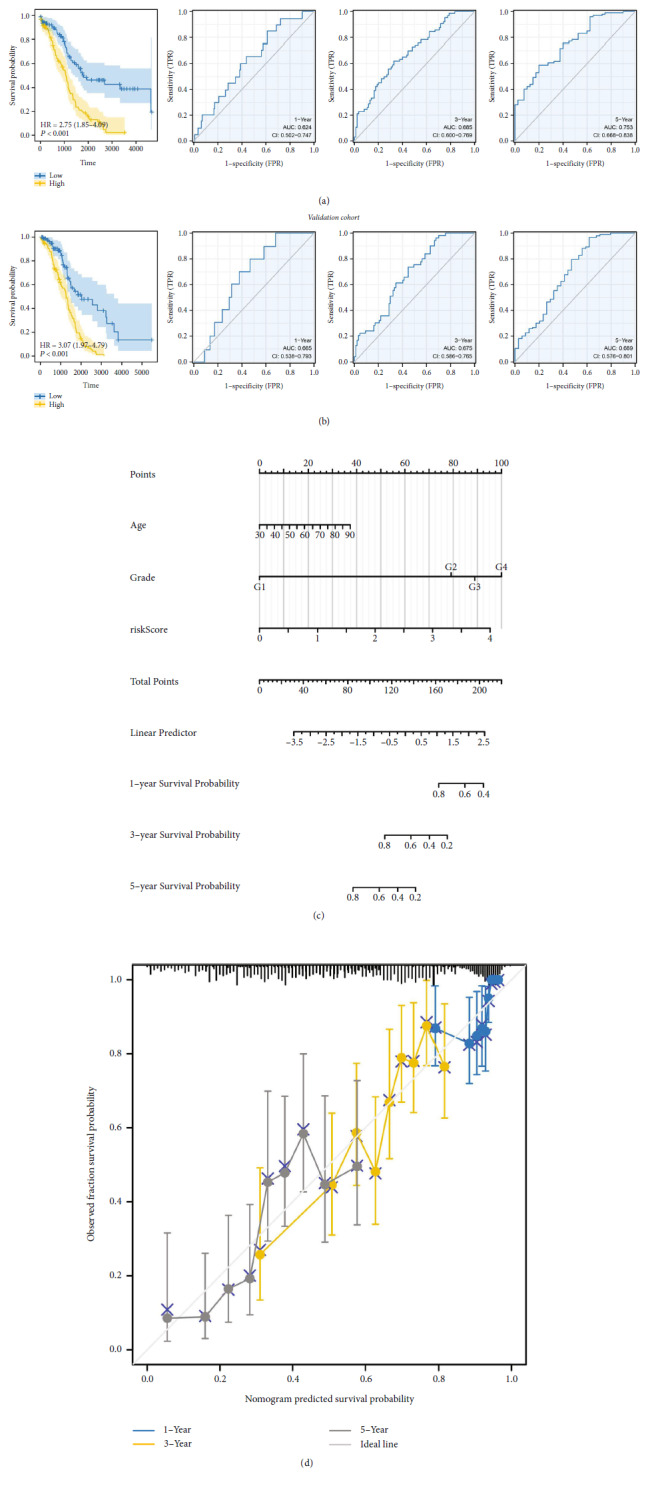
Evaluation of the prognosis model. (a) KM and ROC curves of our model in training cohort. (b) KM and ROC curves of our model in validation cohort. (c) A nomogram was established by combining the risk score and clinical features. (d) Calibration plots.

Our training cohort showed that patients with a high-risk score may have a worse survival rate (Figure 4(a)). ROC curves presented a satisfactory prediction efficiency of our signature on patients survival (Figure 4(a); the area under the curve (AUC) value of 1-, 3-, and 5-year survival were 0.624, 0.685, and 0.753). The same result was also observed in the validation cohort (Figure 4(b); the AUC value of 1-, 3-, and 5-year survival were 0.665, 0.675, and 0.689). A nomogram was constructed by combining the risk score and clinical features to better predict patients survival (Figure 4(c)). The calibration curve indicated a good fit between the actual and nomogram-predicted survival (Figure 4(d)).

### 3.4. Microenvironment Quantification

**Figure 5:**
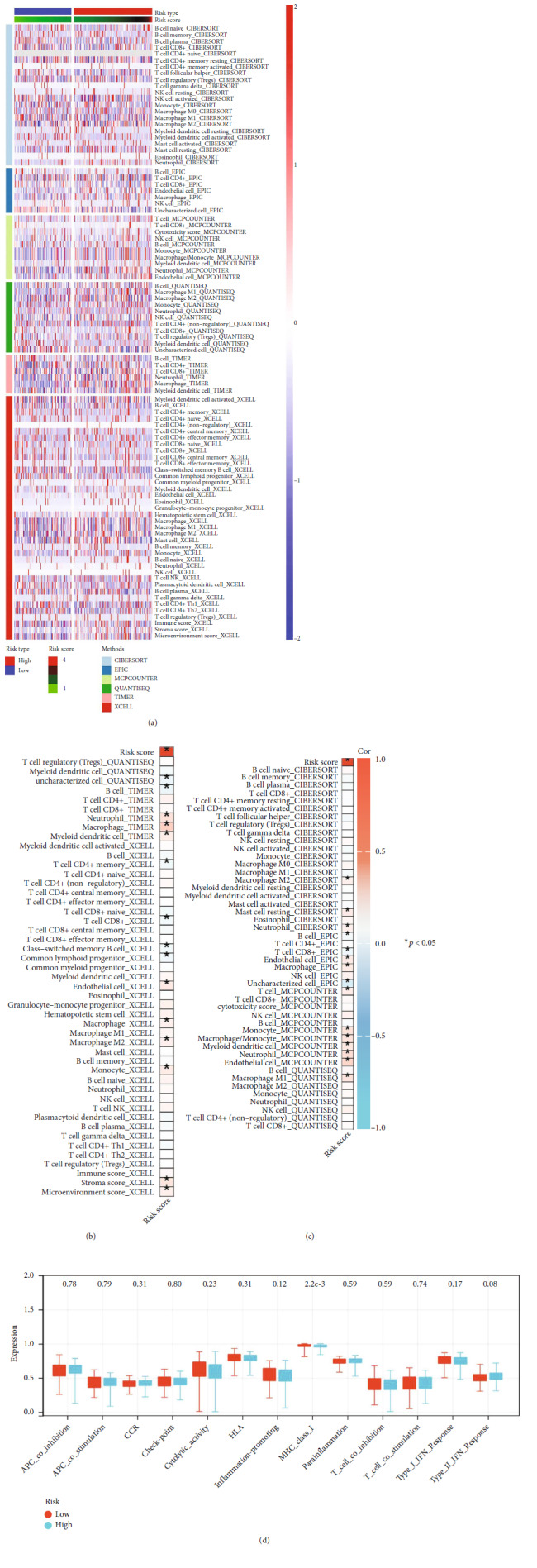
Immune microenvironment difference between high and low risk patients. (a) The tumor microenvironment of OC was quantified using the multiple algorithm. (b and c) Correlation between risk score and quantified cells. (d) The level of immune function in high- and low-risk patients.

We next quantified the cell infiltration of OC patients using multiple algorithms, including CIBERSORT, EPIC, MCP-counter, quanTIseq, TIMER, and xCell (Figure 5(a)). Results indicated that the risk score was positively correlated with neutrophils, macrophages, monocyte, myeloid dendritic cells, and endothelial cells, whereas negatively correlated with B cells and CD8-positive T-lymphocytes (CD8+ T) cells (Figures 5(b) and 5(c)). Immune function analysis showed that the high-risk patients might have a lower activity of major histocompatibility complex (MHC) class I (Figure 5(d)).

### 3.5. Evaluation of Immunotherapy and Drug Sensitivity

**Figure 6:**
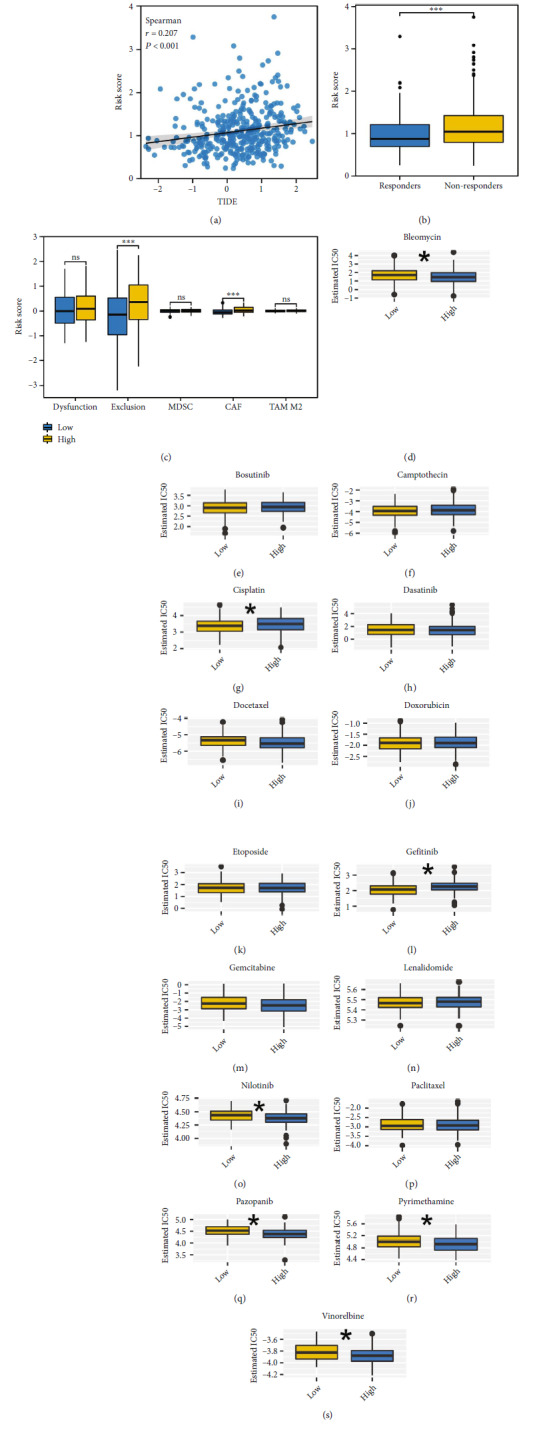
Immunotherapy and drug sensitivity analysis. (a) Correlation between the risk score and TIDE score. (b) The level of risk score in immunotherapy responders and non-responders. (c) The level of dysfunction, exclusion, MDSC, CAF, and TAM M2 in high- and low-risk patients. (d–s) Drug sensitivity analysis.

We next evaluated the immunotherapy sensitivity differences. The result indicated a positive correlation between the risk score and the TIDE score (Figure 6(a), *r* = 0.207, *P* < 0.001). Meanwhile, we noticed that the immunotherapy non-responders might have a higher risk score (Figure 6(b)). Moreover, we noticed a higher level of immune exclusion and Carcinoma-associated fibroblasts (CAFs) infiltration in high-risk patients (Figure 6(c)). Drug sensitivity analysis indicated that high-risk patients might respond better to bleomycin, nilotinib, pazopanib, pyrimethamine, and vinorelbine, yet resistant to cisplatin and gefitinib (Figures 6(d), 6(e), 6(f), 6(g), 6(h), 6(i), 6(j), 6(k), 6(l), 6(m), 6(n), 6(o), 6(p), 6(q), 6(r), and 6(s)).

### 3.6. Biological Enrichment Analysis

**Figure 7:**
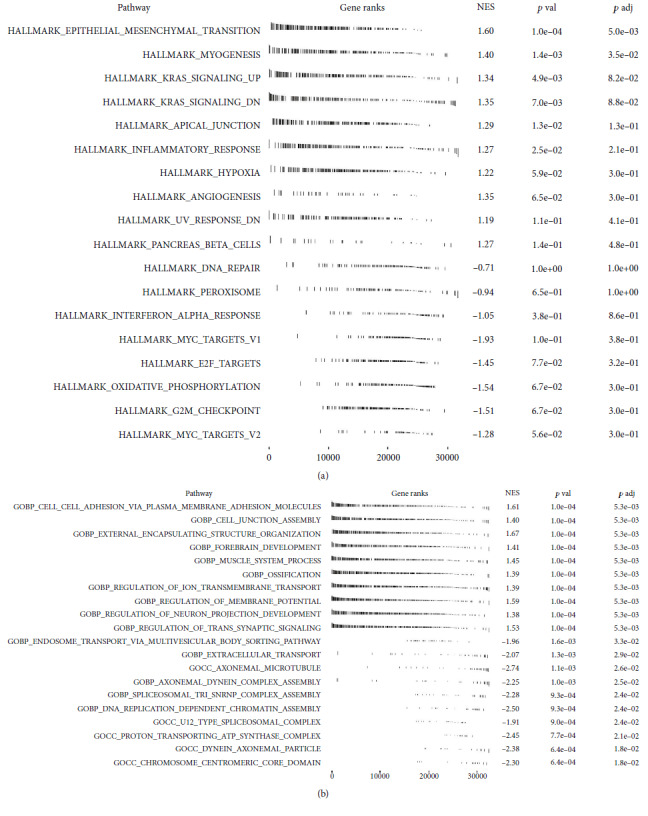
Biological enrichment analysis. (a) GSEA analysis based on Hallmark gene set. (b) GSEA analysis based on GO gene set.

The GSEA analysis based on the Hallmark gene set indicated that the pathways of EMT, myogenesis, KRAS signaling, apical junction, and inflammatory response were activated in high-risk patients (Figure 7(a)). The GSEA analysis based on the GO gene set showed that the terms of external encapsulating structure organization, forebrain development, and muscle system process were activated (Figure 7(b)).

### 3.7. Further Investigation of ECH1

**Figure 8:**
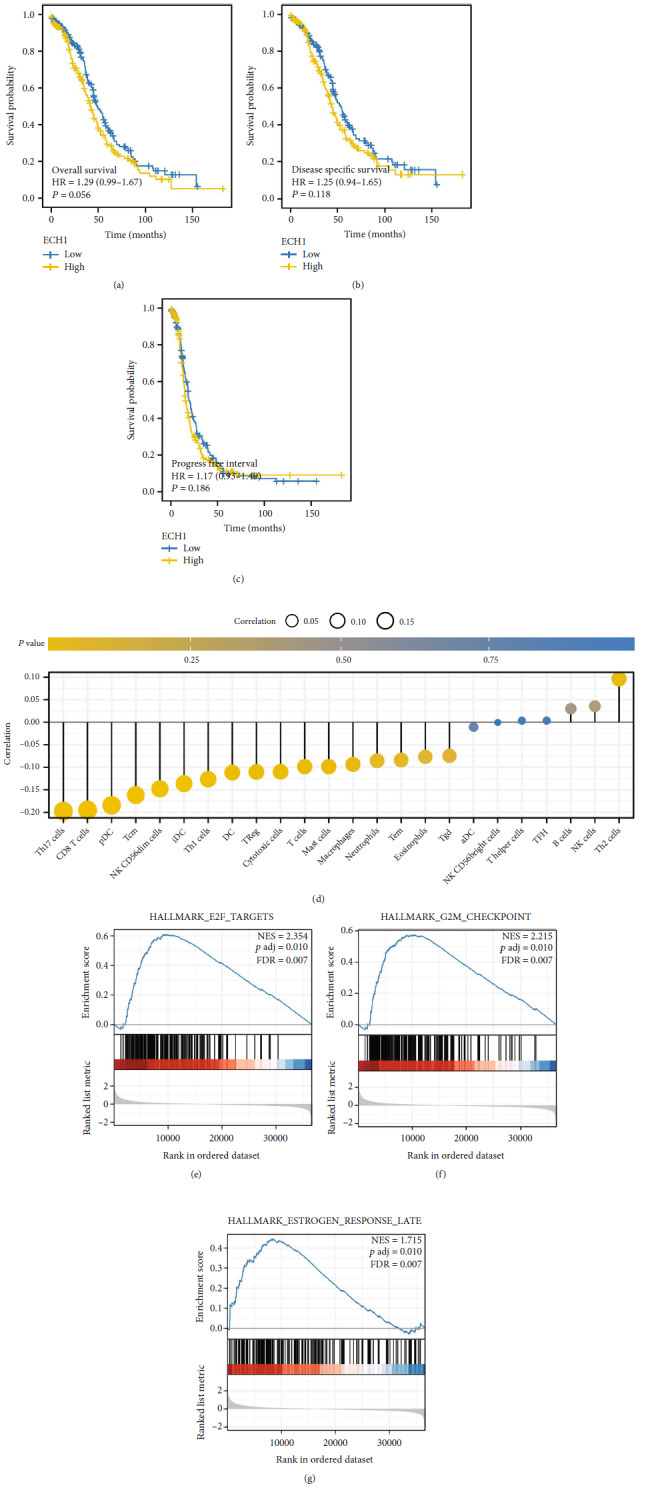
Further exploration of the ECH1. (a–c) KM survival curves of ECH1 (OS, DSS, and PFI). (d) Immune cell correlation of ECH1. (e–g): The top 3 enriched Hallmark pathways.

The ECH1 was then selected for further analysis. Although not statistically significant, considering the significant difference between KM curves, we believed that the patients with high ECH1 tend to have a worse prognosis (Figures 8(a), 8(b), and 8(c)). Immune infiltration analysis showed that ECH1 was positively correlated with Th2 cells, yet negatively correlated with T helper cell 17 (Th17) cells, CD8+ T cells, plasmacytoid DC (pDC), Central Memory T cell (Tcm), and CD56dim NK cells (Figure 8(d)). GSEA analysis indicated that the top 3 pathways ECH1 was involved in were E2F targets, G2M checkpoints, and estrogen response late (Figures 8(e), 8(f), and 8(g)).

## 4. Discussion

OC remains the primary threat to women's health globally [21]. OC often occurs in perimenopausal women. Due to the lack of early symptoms and effective diagnostic methods, the mortality of OC ranks first among gynecological malignancies [22]. Moreover, the recurrence of OC can be considered a fatal chronic disease with limited treatment. Therefore, exploring its internal mechanism from a molecular perspective can effectively promotes the clinical application of OC.

The development of bioinformatics provides us with an opportunity to deeply understand the mechanism of disease [23]. In our study, we comprehensively investigated the PPAR target genes in OC, including their biological role. Meanwhile, a prognosis signature consisting of eight PPAR target genes was established, including APOA5, UGT2B4, TSC22D1, GHITM, REN, DOCK4, ECH1, and ANGPTL4. A nomogram was constructed by combining the clinical feature and risk score. Immune infiltration and biological enrichment analysis were applied to investigate the difference between high- and low-risk patients. Immunotherapy and drug sensitivity analysis were then conducted. Furthermore, the gene ECH1 was selected for further analysis.

Our prognosis signature consists of eight PPAR genes, including APOA5, UGT2B4, TSC22D1, GHITM, REN, DOCK4, ECH1, and ANGPTL4. Some studies have explored their role in cancers. For instance, the polymorphisms of UGT2B4 were reported to be associated with pancreatic cancer, breast cancer, and esophageal cancer [24–26]. In breast cancer, Meijer et al. found that the TSC22D1 could predict the clinical outcome of patients treated with tamoxifen [27]. Zhao et al. noticed that DOCK4 is a biomarker indicating the prognosis and sensitivity to platinum [28]. Kobayashi et al. revealed that the complex formed by DOCK4 and SH3YL1 could induce Rac1 activation and promote cell migration [29]. Zhang et al. found that ECH1 is an effective inhibitor for lymphatic metastasis of liver cancer [30]. Hui et al. noticed that the long non-coding RNA (lncRNA) AGAP2-AS1 induced by RREB1 could affect the malignant behaviors of pancreatic cancer by suppressing the ankyrin repeat domain 1 and ANGPTL4 [31]. Our results present the role of these genes in OC, which can provide direction for future studies.

GSEA analysis indicated that the pathways of the inflammatory response, EMT, myogenesis, and KRAS signaling were activated in high-risk patients. Liang et al. indicated that in OC, by competitively binding miR-101-3p, lncRNA PTAR promotes EMT and invasion-metastasis [32]. Wu et al. showed that ST3GAL1 could facilitate OC cancer progression through EMT signaling [33]. Kim et al. indicated that the silence of the KRAS gene could indicate a novel treatment strategy for OC [34]. Our result indicated that the poor prognosis of high-risk patients might be due to the abnormal activation of these pathways.

Results indicated that the risk score was positively correlated with neutrophils, macrophages, monocyte, myeloid dendritic cells, and endothelial cells, whereas negatively correlated with B cells and CD8+ T cells. Endothelial cells could promote angiogenesis in the tumor microenvironment, which is a key factor in tumor metastasis [35]. In OC, Li et al. found that the chemoresistant OC cells could promote angiogenesis through exosome manners [36]. Macrophages have also been found to exert an important role in OC. For example, Song et al. noticed that the ubiquitin protein ligase E3 component n-recognin 5 derived from the immunosuppressive macrophages could facilitate the OC progression [37]. Zeng et al. demonstrated that the EGF secreted by the M2 macrophages could enhance OC metastasis by activating epidermal growth factor receptor–extracellular regulated protein kinases signaling and inhibiting the expression of lncRNA LIMIT [38]. Muthuswamy et al. noticed that the CXCR6 could promote immunosurveillance and control in the OC microenvironment through increasing the retention of memory CD8+ T cells [39]. Our results indicated that the diverse immune cell infiltration pattern can be partly responsible for the difference in prognosis.

ECH1 was selected for our further analysis. Previous studies have shown its role in cancers. Zhang et al. revealed that ECH1 is a potent inhibitor in the process of lymphatic metastasis in liver cancer [30]. Dai et al. found that the ECH1 and HNRNPA2B1 could be a biomarkers for the early diagnosis of lung cancer [40]. Our study illustrated the role of ECH1 in OC, which could provide direction for follow-up research.

Some limitations should be noticed. First, since most of the patients included are from Western populations, this study is inevitably affected by race bias. Second, the results of bioinformatics can not directly reflect the real biological role. Consequently, further biological validation is necessary for the future.

## Figures and Tables

**Table 1:** The baseline information of the enrolled patients.

Clinical features		Number	Percentage (%)
Age (years)	≤60	326	55.5
	>60	261	44.5
Grade	G1–G2	75	12.8
	G3–G4	496	84.5
	Unknown	16	2.7

## Data Availability

Data supporting this research article are available from the corresponding author or first author on reasonable request.

## Supplementary Materials

Supplementary Materials Figure S1. The biological enrichment analysis of APOA5, UGT2B4, TSC22D1, GHITM, REN, DOCK4, ECH1, and ANGPTL4. (a) GSEA analysis of ANGPTL4 based on Hallmark gene set. (b) GSEA analysis of APOA5 based on Hallmark gene set. (c) GSEA analysis of DOCK4 based on Hallmark gene set. (d) GSEA analysis of ECH1 based on Hallmark gene set. (e) GSEA analysis of GHITM based on Hallmark gene set. (f) GSEA analysis of REN based on Hallmark gene set. (g) GSEA analysis of TSC22D1 based on Hallmark gene set. (h) GSEA analysis of UGT2B4 based on Hallmark gene set. Table S1. The list of PPAR-related genes.
