# Supplementary material for: Comprehensive Analysis Identifies the PPAR-Targeted Genes Associated with Ovarian Cancer Prognosis and Tumor Microenvironment
Source: PPAR Res. 2023 May 11;2023:6637414. doi: 10.1155/2023/6637414 (PMC10195182; doi:10.1155/2023/6637414)
Supplement: Supplementary Materials — Figure S1. The biological enrichment analysis of APOA5, UGT2B4, TSC22D1, GHITM, REN, DOCK4, ECH1, and ANGPTL4. (a) GSEA analysis of ANGPTL4 based on Hallmark gene set. (b) GSEA analysis of APOA5 based on Hallmark gene set. (c) GSEA analysis of DOCK4 based on Hallmark gene set. (d) GSEA analysis of ECH1 based on Hallmark gene set. (e) GSEA analysis of GHITM based on Hallmark gene set. (f) GSEA analysis of REN based on Hallmark gene set. (g) GSEA analysis of TSC22D1 based on Hallmark gene set. (h) GSEA analysis of UGT2B4 based on Hallmark gene set. Table S1. The list of PPAR-related genes. [file 6637414.f1.pdf]

Supplementary file

Figure S1

A ANGPTL4

Pathway	NES	pval	padj
HALLMARK_ANGIOGENESIS	2.29	2.4e−04	6.7e−04
HALLMARK_CHOLESTEROL_HOMEOSTASIS	2.03	2.7e−04	6.7e−04
HALLMARK_IL6_JAK_STAT3_SIGNALING	1.90	2.7e−04	6.7e−04
HALLMARK_COAGULATION	1.78	3.0e−04	6.7e−04
HALLMARK_UV_RESPONSE_DN	2.32	3.0e−04	6.7e−04
HALLMARK_FATTY_ACID_METABOLISM	1.83	3.0e−04	6.7e−04
HALLMARK_UV_RESPONSE_UP	1.88	3.0e−04	6.7e−04
HALLMARK_ADIPOGENESIS	2.18	3.2e−04	6.7e−04
HALLMARK_MITOTIC_SPINDLE	1.92	3.2e−04	6.7e−04
HALLMARK_MYOGENESIS	2.23	3.2e−04	6.7e−04
HALLMARK_DNA_REPAIR	−0.66	1.0e+00	1.0e+00
HALLMARK_OXIDATIVE_PHOSPHORYLATION	−0.87	7.9e−01	9.0e−01
HALLMARK_SPERMATOGENESIS	−1.18	1.5e−01	1.8e−01
HALLMARK_MYC_TARGETS_V1	−1.18	1.3e−01	1.7e−01
HALLMARK_INTERFERON_ALPHA_RESPONSE	−1.41	2.7e−02	3.9e−02

Gene ranks: 0 10000 20000 30000 40000 50000

B APOA5

Pathway	NES	pval	padj
HALLMARK_KRAS_SIGNALING_DN	1.83	1.4e−04	2.0e−03
HALLMARK_SPERMATOGENESIS	1.55	4.8e−03	1.4e−02
HALLMARK_PANCREAS_BETA_CELLS	1.56	2.3e−02	5.1e−02
HALLMARK_HEDGEHOG_SIGNALING	1.45	5.6e−02	1.1e−01
HALLMARK_WNT_BETA_CATENIN_SIGNALING	1.16	2.4e−01	3.8e−01
HALLMARK_BILE_ACID_METABOLISM	1.07	3.4e−01	4.9e−01
HALLMARK_MITOTIC_SPINDLE	1.01	4.4e−01	6.2e−01
HALLMARK_E2F_TARGETS	1.01	4.5e−01	6.2e−01
HALLMARK_G2M_CHECKPOINT	0.98	5.0e−01	6.7e−01
HALLMARK_MYOGENESIS	0.96	5.5e−01	7.2e−01
HALLMARK_IL2_STAT5_SIGNALING	−1.61	7.3e−04	3.3e−03
HALLMARK_ADIPOGENESIS	−1.64	7.3e−04	3.3e−03
HALLMARK_KRAS_SIGNALING_UP	−2.00	3.6e−04	2.0e−03
HALLMARK_INFLAMMATORY_RESPONSE	−2.15	3.6e−04	2.0e−03
HALLMARK_TNFA_SIGNALING_VIA_NFKB	−2.40	3.6e−04	2.0e−03
HALLMARK_OXIDATIVE_PHOSPHORYLATION	−1.93	3.6e−04	2.0e−03
HALLMARK_INTERFERON_GAMMA_RESPONSE	−2.11	3.6e−04	2.0e−03
HALLMARK_EPITHELIAL_MESENCHYMAL_TRANSITION	−2.52	3.6e−04	2.0e−03
HALLMARK_COMPLEMENT	−1.92	3.6e−04	2.0e−03
HALLMARK_ALLOGRAFT_REJECTION	−1.99	3.6e−04	2.0e−03

Gene ranks: 0 10000 20000 30000 40000 50000

C DOCK4

Pathway	NES	pval	padj
HALLMARK_ANGIOGENESIS	2.67	4.0e−04	2.5e−03
HALLMARK_HEDGEHOG_SIGNALING	2.28	4.0e−04	2.5e−03
HALLMARK_TGF_BETA_SIGNALING	2.42	4.7e−04	2.5e−03
HALLMARK_IL6_JAK_STAT3_SIGNALING	2.53	6.2e−04	2.5e−03
HALLMARK_COAGULATION	2.28	8.3e−04	2.5e−03
HALLMARK_WNT_BETA_CATENIN_SIGNALING	2.01	8.6e−04	2.5e−03
HALLMARK_UV_RESPONSE_DN	3.17	8.8e−04	2.5e−03
HALLMARK_APOPTOSIS	1.74	1.0e−03	2.5e−03
HALLMARK_ALLOGRAFT_REJECTION	2.89	1.2e−03	2.5e−03
HALLMARK_INTERFERON_GAMMA_RESPONSE	1.49	1.2e−03	2.5e−03
HALLMARK_INTERFERON_ALPHA_RESPONSE	−0.85	7.4e−01	8.3e−01
HALLMARK_FATTY_ACID_METABOLISM	−0.96	5.7e−01	6.7e−01
HALLMARK_REACTIVE_OXYGEN_SPECIES_PATHWAY	−0.97	5.1e−01	6.2e−01
HALLMARK_PEROXISOME	−1.01	4.5e−01	5.6e−01
HALLMARK_MYC_TARGETS_V2	−1.08	3.5e−01	4.7e−01
HALLMARK_PANCREAS_BETA_CELLS	−1.15	2.7e−01	3.7e−01
HALLMARK_SPERMATOGENESIS	−1.34	4.3e−02	6.9e−02
HALLMARK_DNA_REPAIR	−1.42	1.7e−02	2.9e−02
HALLMARK_MYC_TARGETS_V1	−1.57	8.7e−04	2.5e−03
HALLMARK_OXIDATIVE_PHOSPHORYLATION	−2.33	1.1e−04	2.5e−03

Gene ranks: 0 10000 20000 30000 40000 50000

D ECH1

Pathway	NES	pval	padj
HALLMARK_KRAS_SIGNALING_DN	2.04	1.3e−04	1.7e−03
HALLMARK_E2F_TARGETS	2.32	1.3e−04	1.7e−03
HALLMARK_G2M_CHECKPOINT	2.20	1.3e−04	1.7e−03
HALLMARK_SPERMATOGENESIS	2.27	1.4e−04	1.7e−03
HALLMARK_MITOTIC_SPINDLE	1.67	2.6e−04	2.3e−03
HALLMARK_PANCREAS_BETA_CELLS	1.93	3.2e−04	2.3e−03
HALLMARK_GLYCOLYSIS	1.55	1.3e−03	5.8e−03
HALLMARK_MTORC1_SIGNALING	1.52	3.1e−03	1.2e−02
HALLMARK_HEDGEHOG_SIGNALING	1.72	4.8e−03	1.6e−02
HALLMARK_DNA_REPAIR	1.51	6.7e−03	2.1e−02
HALLMARK_INTERFERON_ALPHA_RESPONSE	−1.11	2.2e−01	3.2e−01
HALLMARK_APOPTOSIS	−1.15	1.3e−01	2.2e−01
HALLMARK_UV_RESPONSE_DN	−1.25	5.6e−02	1.0e−01
HALLMARK_ALLOGRAFT_REJECTION	−1.30	2.0e−02	4.9e−02
HALLMARK_ANGIOGENESIS	−1.77	3.5e−03	1.2e−02
HALLMARK_COAGULATION	−1.53	1.9e−03	8.1e−03
HALLMARK_TNFA_SIGNALING_VIA_NFKB	−2.07	4.7e−04	2.3e−03
HALLMARK_EPITHELIAL_MESENCHYMAL_TRANSITION	−2.16	4.7e−04	2.3e−03
HALLMARK_INFLAMMATORY_RESPONSE	−1.77	4.6e−04	2.3e−03
HALLMARK_INTERFERON_GAMMA_RESPONSE	−1.61	4.6e−04	2.3e−03

Gene ranks: 0 10000 20000 30000 40000 50000

E GHITM

Pathway	NES	pval	padj
HALLMARK_REACTIVE_OXYGEN_SPECIES_PATHWAY	1.90	3.0e−03	9.4e−02
HALLMARK_MYC_TARGETS_V2	1.64	4.2e−03	9.4e−02
HALLMARK_CHOLESTEROL_HOMEOSTASIS	1.65	7.0e−03	9.4e−02
HALLMARK_ANGIOGENESIS	1.65	1.3e−02	9.4e−02
HALLMARK_INTERFERON_ALPHA_RESPONSE	1.87	1.4e−02	9.4e−02
HALLMARK_PROTEIN_SECRETION	2.33	1.4e−02	9.4e−02
HALLMARK_PI3K_AKT_MTOR_SIGNALING	1.48	1.7e−02	9.4e−02
HALLMARK_ANDROGEN_RESPONSE	2.20	1.8e−02	9.4e−02
HALLMARK_PEROXISOME	1.92	1.8e−02	9.4e−02
HALLMARK_UNFOLDED_PROTEIN_RESPONSE	2.07	1.9e−02	9.4e−02
HALLMARK_COAGULATION	−0.67	9.8e−01	9.8e−01
HALLMARK_APICAL_SURFACE	−0.57	9.8e−01	9.8e−01
HALLMARK_MYOGENESIS	−0.75	9.6e−01	9.8e−01
HALLMARK_WNT_BETA_CATENIN_SIGNALING	−0.64	9.5e−01	9.8e−01
HALLMARK_TGF_BETA_SIGNALING	−0.81	8.0e−01	8.7e−01

Gene ranks: 0 10000 20000 30000 40000 50000

F REN

Pathway	NES	pval	padj
HALLMARK_ESTROGEN_RESPONSE_LATE	1.63	2.6e−04	2.6e−03
HALLMARK_KRAS_SIGNALING_DN	1.77	2.6e−04	2.6e−03
HALLMARK_G2M_CHECKPOINT	1.57	7.7e−04	4.8e−03
HALLMARK_ESTROGEN_RESPONSE_EARLY	1.55	7.7e−04	4.8e−03
HALLMARK_APICAL_JUNCTION	1.55	7.8e−04	4.8e−03
HALLMARK_COAGULATION	1.56	2.8e−03	1.5e−02
HALLMARK_E2F_TARGETS	1.45	9.0e−03	4.5e−02
HALLMARK_APICAL_SURFACE	1.63	1.3e−02	6.0e−02
HALLMARK_NOTCH_SIGNALING	1.64	1.6e−02	6.6e−02
HALLMARK_MYOGENESIS	1.25	5.5e−02	1.9e−01
HALLMARK_IL2_STAT5_SIGNALING	−0.84	8.8e−01	1.0e+00
HALLMARK_APOPTOSIS	−0.84	8.4e−01	1.0e+00
HALLMARK_ADIPOGENESIS	−0.88	7.9e−01	1.0e+00
HALLMARK_TNFA_SIGNALING_VIA_NFKB	−1.00	4.5e−01	7.7e−01
HALLMARK_ANGIOGENESIS	−1.05	3.8e−01	6.7e−01
HALLMARK_INFLAMMATORY_RESPONSE	−1.04	3.5e−01	6.5e−01
HALLMARK_IL6_JAK_STAT3_SIGNALING	−1.36	4.7e−02	1.8e−01
HALLMARK_INTERFERON_ALPHA_RESPONSE	−1.99	1.7e−04	2.6e−03
HALLMARK_ALLOGRAFT_REJECTION	−1.88	1.6e−04	2.6e−03
HALLMARK_INTERFERON_GAMMA_RESPONSE	−2.07	1.6e−04	2.6e−03

Gene ranks: 0 10000 20000 30000 40000 50000

G TSC22D1

Pathway	NES	pval	padj
HALLMARK_ANGIOGENESIS	2.03	2.7e−04	1.2e−03
HALLMARK_HEDGEHOG_SIGNALING	2.15	2.7e−04	1.2e−03
HALLMARK_WNT_BETA_CATENIN_SIGNALING	2.32	2.8e−04	1.2e−03
HALLMARK_TGF_BETA_SIGNALING	2.39	2.9e−04	1.2e−03
HALLMARK_COAGULATION	1.92	3.5e−04	1.2e−03
HALLMARK_UV_RESPONSE_DN	2.45	3.5e−04	1.2e−03
HALLMARK_EPITHELIAL_MESENCHYMAL_TRANSITION	3.25	4.0e−04	1.2e−03
HALLMARK_ESTROGEN_RESPONSE_EARLY	1.88	4.0e−04	1.2e−03
HALLMARK_TNFA_SIGNALING_VIA_NFKB	2.70	4.0e−04	1.2e−03
HALLMARK_APICAL_JUNCTION	2.29	4.0e−04	1.2e−03
HALLMARK_ADIPOGENESIS	−1.16	1.6e−01	2.3e−01
HALLMARK_ALLOGRAFT_REJECTION	−1.19	1.2e−01	1.8e−01
HALLMARK_DNA_REPAIR	−1.28	7.0e−02	1.1e−01
HALLMARK_REACTIVE_OXYGEN_SPECIES_PATHWAY	−1.48	2.9e−02	4.6e−02
HALLMARK_INTERFERON_GAMMA_RESPONSE	−1.54	1.9e−03	3.9e−03
HALLMARK_FATTY_ACID_METABOLISM	−1.64	8.2e−04	2.0e−03
HALLMARK_MYC_TARGETS_V1	−1.60	8.0e−04	2.0e−03
HALLMARK_INTERFERON_ALPHA_RESPONSE	−1.79	1.5e−04	1.2e−03
HALLMARK_OXIDATIVE_PHOSPHORYLATION	−2.37	1.3e−04	1.2e−03
HALLMARK_E2F_TARGETS	−1.86	1.3e−04	1.2e−03

Gene ranks: 0 10000 20000 30000 40000 50000

H UGT2B4

Pathway	NES	pval	padj
HALLMARK_EPITHELIAL_MESENCHYMAL_TRANSITION	2.35	1.5e−04	5.3e−03
HALLMARK_MITOTIC_SPINDLE	1.46	4.2e−03	3.2e−02
HALLMARK_UV_RESPONSE_DN	1.52	4.5e−03	3.2e−02
HALLMARK_TNFA_SIGNALING_VIA_NFKB	1.45	6.7e−03	3.7e−02
HALLMARK_SPERMATOGENESIS	1.50	8.1e−03	4.0e−02
HALLMARK_TGF_BETA_SIGNALING	1.62	1.0e−02	4.8e−02
HALLMARK_ANGIOGENESIS	1.63	1.2e−02	5.0e−02
HALLMARK_COAGULATION	1.30	6.3e−02	2.0e−01
HALLMARK_KRAS_SIGNALING_UP	1.25	7.0e−02	2.1e−01
HALLMARK_INFLAMMATORY_RESPONSE	1.17	1.6e−01	3.6e−01
HALLMARK_BILE_ACID_METABOLISM	−1.24	8.9e−02	2.3e−01
HALLMARK_XENOBIOTIC_METABOLISM	−1.21	7.6e−02	2.1e−01
HALLMARK_FATTY_ACID_METABOLISM	−1.29	5.1e−02	1.7e−01
HALLMARK_DNA_REPAIR	−1.31	3.9e−02	1.4e−01
HALLMARK_PEROXISOME	−1.50	1.4e−02	5.2e−02
HALLMARK_ADIPOGENESIS	−1.42	6.3e−03	3.7e−02
HALLMARK_ESTROGEN_RESPONSE_EARLY	−1.52	2.9e−03	2.9e−02
HALLMARK_REACTIVE_OXYGEN_SPECIES_PATHWAY	−1.81	4.9e−04	6.1e−03
HALLMARK_OXIDATIVE_PHOSPHORYLATION	−2.63	3.2e−04	5.3e−03
HALLMARK_ESTROGEN_RESPONSE_LATE	−1.71	3.2e−04	5.3e−03

Gene ranks: 0 10000 20000 30000 40000 50000

Table S1. The list of PPAR-related genes
GYS2, HMOX1, SLC25A20, NPC1L1, SLC29A1, DBI, UCP3, FABP6, PLIN2, CPT1B, APOA1, AHR, CYP1A1, UGDH, ACOX1, GPT, FADS2, PDZK1, CREB3L3, KLF10, KLF11, MAP3K8, APOA5, C3, CYP7A1, ACADM, TXN, ABCG2, TF, RETSAT, NR1D1, SULT2A1, AP2A2, CPT2, UGT1A9, UGT2B4, CYP3A4, ASS1, TSC22D1, GPD1, G0S2, HCAR1, NAMPT, INSR, TFF2, PGK1, PKM, APOE, PTGS2, LRP1, SCARB1, SGK1, BRCA1, SAT1, BCL2, CAV1, CYP27A1, GHITM, VEGFA, TXNIP, SDC1, REN, CDKN1A, SHBG, GCK, SLC9A1, CAT, SLC22A5, CTSL1, TNFSF10, TNIP1, KLF4, IRF1, AK3, APH1B, CTBS, CYB5D2, DIAPH1, DOCK4, ECH1, ETFB, HSDL2, IMPA2, ISCA1, MLYCD, MYO18A, NUDT13, NUDT9, PPARG, RUNX1, SYTL3, TIMP4, TTC33, UROS, ZNF354A, ZNF367, ABCA1, ACSL5, ANGPTL4, AP2A1, ETFDH, PDK4, PLIN1, TMEM135, VAMP8, AHNAK, BIRC3, CDKN2C, BLOC1S4, CSNK1G2, DCP1A, EXOC6B, GPR180, GRAMD3, IFIT2, TALDO1, TNFRSF1A, PDK2, PDK3, HBEGF, APOA2, INSIG1, YWHAE, FABP5, ACSL3, LDLR
